# Causal Inferences in Repetitive Transcranial Magnetic Stimulation Research: Challenges and Perspectives

**DOI:** 10.3389/fnhum.2020.586448

**Published:** 2021-01-14

**Authors:** Justyna Hobot, Michał Klincewicz, Kristian Sandberg, Michał Wierzchoń

**Affiliations:** ^1^Consciousness Lab, Psychology Institute, Jagiellonian University, Krakow, Poland; ^2^Center of Functionally Integrative Neuroscience, Aarhus University, Aarhus, Denmark; ^3^Cognitive Science, Institute of Philosophy, Jagiellonian University, Krakow, Poland; ^4^Department of Cognitive Science and Artificial Intelligence, Tilburg University, Tilburg, Netherlands; ^5^Center of Functionally Integrative Neuroscience, Aarhus University Hospital, Aarhus, Denmark

**Keywords:** causal inferences, brain plasticity, brain excitability, repetitive TMS, TMS-neuroimaging

## Abstract

Transcranial magnetic stimulation (TMS) is used to make inferences about relationships between brain areas and their functions because, in contrast to neuroimaging tools, it modulates neuronal activity. The central aim of this article is to critically evaluate to what extent it is possible to draw causal inferences from repetitive TMS (rTMS) data. To that end, we describe the logical limitations of inferences based on rTMS experiments. The presented analysis suggests that rTMS alone does not provide the sort of premises that are sufficient to warrant strong inferences about the direct causal properties of targeted brain structures. Overcoming these limitations demands a close look at the designs of rTMS studies, especially the methodological and theoretical conditions which are necessary for the functional decomposition of the relations between brain areas and cognitive functions. The main points of this article are that TMS-based inferences are limited in that stimulation-related causal effects are not equivalent to structure-related causal effects due to TMS side effects, the electric field distribution, and the sensitivity of neuroimaging and behavioral methods in detecting structure-related effects and disentangling them from confounds. Moreover, the postulated causal effects can be based on indirect (network) effects. A few suggestions on how to manage some of these limitations are presented. We discuss the benefits of combining rTMS with neuroimaging in experimental reasoning and we address the restrictions and requirements of rTMS control conditions. The use of neuroimaging and control conditions allows stronger inferences to be gained, but the strength of the inferences that can be drawn depends on the individual experiment’s designs. Moreover, in some cases, TMS might not be an appropriate method of answering causality-related questions or the hypotheses have to account for the limitations of this technique. We hope this summary and formalization of the reasoning behind rTMS research can be of use not only for scientists and clinicians who intend to interpret rTMS results causally but also for philosophers interested in causal inferences based on brain stimulation research.

## Introduction

A fundamental issue in human neuroscience is how to make causal inferences based on research data. Traditional use of neuroimaging methods limits experimental conclusions to correlational inferences (though, the methods of effective connectivity are used to postulate causal inferences; see Valdes-Sosa et al., 2011). Following their introduction, brain stimulation methods, especially TMS, started to be considered as a remedy for this limitation. TMS was developed over thirty years ago and is based on electromagnetic induction (Barker et al., 1985). A TMS coil induces an electric field which might influence the activity of brain tissue. It was originally thought that TMS would make it possible to conclude the causal relations between brain activity, cognitive functions, and behaviors. However, it has since become clearer that the brain cannot simply be parceled into regions responsible for certain functions, and the impact of brain lesions and non-invasive brain stimulation is not necessarily limited to a single area but extends to networks. Currently, TMS is often used to test hypotheses about how short-term changes in the excitability of a stimulated brain area affect cognitive functions. In online TMS paradigms, electromagnetic pulses are applied concurrently with the experimental measurement. The physiological consequences of a single electromagnetic pulse can be detected for over a dozen seconds (Furubayashi et al., 2013). In repetitive (rTMS) paradigms, pulses with a particular frequency pattern are applied during or before experimental measurement because they often lead to neuroplasticity-like changes (Chung et al., 2015). The neuromodulatory rTMS effect can be assessed with standard experimental procedures or neuroimaging techniques (for a review of combined TMS-EEG studies, see Thut and Pascual-Leone, 2010); it can be observed even for up to 45 min after a single protocol application (Huang et al., 2005), or it can last for months after multiple protocol applications over repeated TMS sessions in longitudinal studies (Speer et al., 2000, 2009; Li et al., 2004; Choi et al., 2014, 2019; Kang et al., 2016). Thus, TMS is often considered to be an extension of neuroimaging, which (due to its influence on brain activity) allows causal relations to be tested.

TMS is frequently used to decompose the functional organization of the brain. Multiple scientific articles contain statements that TMS can be used to draw both causal brain-behavior inferences (Sack, 2006; Śliwińska et al., 2014) and causal relationships between brain structure and function (Schutter et al., 2004; Bolognini and Ro, 2010; Hartwigsen, 2015; Veniero et al., 2019). In research practice, this often leads to implicit assumptions that TMS can selectively influence the area of interest, therefore its role can be established. Consequently, multiple studies have presented rTMS-based conclusions on the causal role of certain brain areas in certain cognitive functions (e.g., Carmel et al., 2010; Philiastides et al., 2011; Zanto et al., 2011; Bourgeois et al., 2013; Izuma et al., 2015; Schaal et al., 2015; Siuda-Krzywicka et al., 2016; Montefinese et al., 2017), often without describing alternative explanations or making a distinction between direct and indirect causal effects of an rTMS-induced change in activity in a certain area on a certain behavior or brain process.

Employing chronometry (tracking the time course of functional relevance), online single-pulse, double-pulse, or short-burst TMS protocols (including double-coil approaches) allow investigation of the causal relations between the activity of certain brain areas and behaviors or cognitive functions especially when effective connectivity measures are also employed (e.g., de Graaf et al., 2009). These protocol types might be used to influence cognitive functions or perturb brain activity to track the signal propagation and analyze the topographic pattern of TMS-evoked changes in brain activity. This allows researchers to: (1) identify the brain areas involved in certain behavior; (2) assess the impact of the stimulated brain area upon interconnected areas *via* direct connections or intermediate areas, including inter-hemispheric interactions (Blankenburg et al., 2008); (3) reveal bottom-up and top-down influences between brain areas; and (4) dissect the specific functional contributions of different cortical areas of an investigated network. Crucially, the propagation of TMS-evoked activity can depend on the degree of wakefulness (Massimini et al., 2005), which in some studies may act as a confound but in others may allow the state-dependence of interactions among remote and interconnected brain regions to be investigated. However, this use of TMS is limited to specific experimental designs, and some TMS effects (as in the case of all active TMS protocols) may be side effects of the stimulation procedure (Holmes and Meteyard, 2018; for a review, see Bestmann et al., 2008a).

The rTMS approach is more limited than single-pulse, double-pulse, and burst-pulse TMS in terms of helping to understand the causal relationships between brain areas and cognitive functions

(however, in certain designs rTMS can be used for chronometry, see Rossi et al., 2011). Online rTMS does not allow concurrent brain activity registration using neuroimaging techniques, while offline rTMS effects depend on neuroplasticity-like changes which might occur at various time points after the start or the end of rTMS. Thus, rTMS does not allow tracking of the direct influence of perturbation to determine the time point at which an area makes a critical contribution to a given behavior or to investigate effective connectivity between brain areas. Although most non-invasive stimulation methods share the same limitations as rTMS, for purposes of clarity we narrow the scope of the discussion below to rTMS. Most of the issues, that are mentioned below, related to the pitfalls of TMS have already been selectively discussed (e.g., Siebner and Rothwell, 2003; Robertson et al., 2003; Thickbroom, 2007; Bestmann et al., 2008a; Siebner et al., 2009). The current article aims to combine, organize, and analyze these insights at the theoretical level and indicate their possible consequences for inferences based on rTMS evidence. Below, we first analyze several known methodological issues that can invalidate inferences about direct causal relations between brain areas, brain processes, and cognitive functions investigated with TMS. Second, we discuss the special role that neuroimaging plays in rTMS-based inferences and approaches to creating TMS control conditions.

## Inferences Based on Conditional Statements

Causal inference, and specifically inference based on interventions in the operation of a complex system such as the brain, fall within the theoretical framework of the general theory of causality that was developed by Pearl (2000). We use a small part of Pearl’s Structural Causal Model. This is because unlike causal frameworks such as Bradford Hill’s criteria (Hill, 1965), Pearl’s framework is resistant to counterexamples and makes sense of probabilistic causal inferences about specific mechanisms that are parts of complex systems. In this view, to characterize a relationship between event A and event B as causal is to say that a selective intervention on A might lead to a change in the distribution of B. We assume a causal influence of one event on another is direct if none of the variables included in a given causal model mediates this effect; otherwise, it is indirect. In a setting such as a TMS experiment, where intervention is randomized, we compare the intervention-related distribution of variables with a control distribution and expect to find suitable neuronal candidates that cause the response. For clarity purposes, we address TMS-related inferences with the use of conditional logic.

To consider a simple type of TMS-based inference, assume that a researcher is interested in cognitive function X. To investigate the process (P_X_) that underlies this function, the researcher aims to determine whether brain area 1 (A_1_), which is typically associated with P_X_, is engaged during a task that is assumed to engage cognitive function X (T_X_). For example, one may investigate the involvement of the dorsolateral prefrontal cortex in decision confidence by measuring the effect of rTMS on confidence ratings. In such a case, the hypothesis (H) often states that P_X_ takes place in A_1_ and is tested with the application of an active rTMS protocol 1 (rTMS_1_) to A_1_. We can formally represent this pattern of reasoning in the following way (the logic symbol ∧ represents the logical conjunction, i.e., “and,” and the → represents implication, i.e., “if <antecedent> then <consequent>”):

H – P_X_ takes place in A_1_

S_1A—_rTMS_1_ is applied to A_1_

T_XD—_a difference in T_X_ performance is observed (as compared to a control condition)

$${\text{I}}_{\text{1}}\left(\left(\left(\text{H}\wedge {\text{S}}_{\text{1A}}\right)\to {\text{T}}_{\text{XD}}\right)\wedge \left({\text{S}}_{\text{1A}}\wedge {\text{T}}_{\text{XD}}\right)\right)\to \text{H}$$

Inference 1 (I_1_) states that the statement that P_X_ takes place in A_1_ is true if the following two premises are true: (1) if P_X_ takes place in A_1_ and rTMS_1_ is applied to A_1_ then a difference in T_X_ performance is observed; and (2) rTMS_1_ is applied to A_1_ and a difference in T_X_ performance is observed.

I_1_ depicts the basic form of reasoning used in rTMS research. However, like any inductive inference, this form of reasoning does not always lead to true conclusions. For example, the occurrence of the difference in T_X_ performance may be unrelated to rTMS_1_, in which case, two independent factors contribute to falsely interpreting the consequent of the condition as true. Thus, causal reasoning based on misuse of I_1_ may lead to false conclusions. Possible overconfidence in I_1_-based inferences might also stem from overlooking both how TMS and brains work. First, the assumption that TMS selectively influences a targeted area is not always true. The strength of the induced electric field decreases together with the distance from the coil, so the brain areas above or adjacent to the targeted area are likely to be stimulated more than the intended one (Heller and van Hulsteyn, 1992). Second, applying TMS to one area can indirectly influence multiple brain areas that are structurally connected to it and lead to an alteration of the functional state of the targeted network, as pointed out in several reviews (Ruff et al., 2009; Bolognini and Ro, 2010; Ziemann, 2010; Beynel et al., 2020). In sum, TMS applied to a specific brain region can influence other regions directly (e.g., due to stimulation of an area above or adjacent to the area investigated) or indirectly *via* neural connections (e.g., indirect stimulation of an area that is connected to the investigated area or activity alteration in another area due to excitability alteration in the investigated area). These factors limit the strength of causal conclusions based on I_1_.

**Figure 1:**
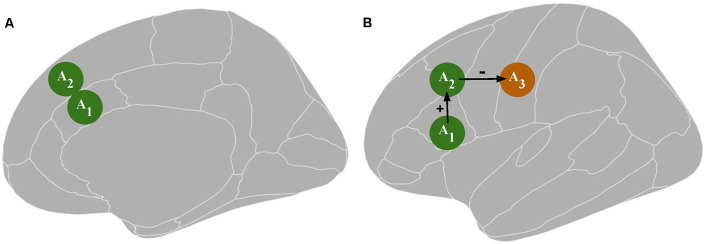
Panel **(A)** depicts a possible direct influence of transcranial magnetic stimulation [TMS; an excitability alteration in the brain tissue surrounding the targeted area A_1_, i.e., area 2 (A_2_)]. Panel **(B)** depicts a possible indirect TMS influence: an excitability alteration in A_2_ or area 3 (A_3_) resulting from an excitability alteration in A_1_. A_1_ represents the targeted area; A_2_ and A_3_ represent the areas directly and indirectly connected to A_1_, respectively, which together constitute a functional network. The green color indicates an increase in neuronal excitation while the orange color indicates a decrease in neuronal excitation.

Accordingly, rTMS_1_ may be responsible for a difference in T_X_ performance *via* unintended stimulation of an area other than A_1_. For example, assume that A_1_ is structurally connected to brain area 2 (A_2_). Then, there is a possibility that A_2_ activity is influenced: (1) directly by rTMS_1_ when A_1_ is targeted (Figure 1A); or (2) indirectly by rTMS_1_ *via* an alteration of A_1_ activity. At the same time, A_2_ is responsible or more important than A_1_ for executing P_X_ (Figure 1B). Unintentional direct stimulation of A_2_ may occur in several ways. First, the physical spread of an electrical field may reach areas adjacent to the targeted one. Second, since electrical current follows the path of least resistance, the electric field distribution is highly dependent on cerebrospinal fluid distribution and brain folding, thus the peak of the electric field can occur in gray matter regions located some distance from the electric field’s expected peak, which is judged based on the location of the center of the (figure-of-eight) coil. This might result in greater stimulation of area/s other than the targeted one (Bijsterbosch et al., 2012). Third, it is challenging to distinguish whether the rTMS effect stems from excitability alteration in the targeted area or an area above it that possibly has a distinct specialization. These concerns may be raised especially when deeper structures such as the anterior cingulate cortex (Hayward et al., 2007) or insula (Pollatos et al., 2016) are investigated. The vast majority of TMS studies target superficial structures; however, the rule that the strongest electrical field is generated within the outermost areas applies even if the distances (which might be the consequences of brain folding) are small. Because a large part of the cortex lies within sulci, targeted brain coordinates in numerous TMS studies have to be placed within sulci (Busan et al., 2009; Cappelletti et al., 2009; Salillas et al., 2009). Additionally, stimulation of deeper brain structures is obtained at the expense of inducing wider electrical field spread in the brain (Roth et al., 2007; Deng et al., 2013; Downar et al., 2016). For example, metabolic and physiological effects on the primary motor cortex and the primary somatosensory cortex can be observed after rTMS to premotor areas (Siebner et al., 2003). This may compound the difficulty in distinguishing the contribution of direct vs. indirect rTMS effects. The network effects may produce remote activity alteration in cortical areas *via* cortico-cortical routes and in subcortical structures *via* cortico-subcortical projections (Strafella et al., 2003; Lefaucheur et al., 2020). The extent of the network effects depends on rTMS protocol parameters (Bestmann et al., 2003). Additionally, the assumption that a difference in T_X_ performance is caused by an rTMS_1_-induced change in A_1_ activity may be misleading due to the occurrence of placebo and sensory side effects (Abler et al., 2005). Moreover, rTMS may influence areas related to general cognitive resources (e.g., regions engaged in attentional or working memory processing) or the observed effect may be specific to the T_X_ design (e.g., resulting from rTMS_1_ influence on brain regions involved in response generation during T_X_), which is not related to the influence on the investigated cognitive function. In sum, overconfidence in I_1_ has multiple ways to lead researchers to overinterpret their data as evidence that P_X_ takes place in A_1_.

Since statements that follow I_1_ cannot fully support the conclusion that P_X_ takes place in A_1_, can some other inference be used to show that P_X_ is not executed in A_1_? This would provide independent evidence for excluding that region from the area of research interest. This way of reasoning is indeed found in TMS literature: based on the lack of an observed effect, some authors postulate a lack of rTMS influence on investigated cognitive functions (e.g., Ghabra et al., 1999; Poulet et al., 2004; Jung et al., 2010; Bor et al., 2017), which might suggest that an investigated area is not involved in the process underlying the investigated cognitive function. Consider then the inference of the following structure (the logic symbol ⇁ represents negation, i.e., “not”):

H – P_X_ takes place in A_1_

S_1A—_rTMS_1_ is applied to A_1_

T_XD—_a difference in T_X_ performance is observed (as compared to a control condition)

$${\text{I}}_{\text{2}}\left(\left(\left(\text{H}\wedge {\text{S}}_{\text{1A}}\right)\to {\text{T}}_{\text{XD}}\right)\wedge \left({\text{S}}_{\text{1A}}\wedge {\text{T}}_{\text{XD}}\right)\right)\to \neg \text{H}$$

Inference 2 (I_2_) states that the statement that P_X_ is not executed in A_1_ is true if the following two premises are also true: (1) a difference in T_X_ performance is observed if P_X_ takes place in A_1_ and rTMS_1_ is applied to A_1_; (2) rTMS_1_ is applied to A_1_ and a difference in T_X_ performance is not observed.

In research practice, rTMS_1_ does not always lead to a change in A_1_ activity and/or a difference in T_X_ performance. rTMS_1_ may have no factual effect because: (1) the rTMS_1_ frequency pattern is inadequate for investigating P_X_ (e.g., theta burst stimulation is applied but P_X_ is independent of theta-gamma coupling; De Ridder et al., 2007); (2) rTMS_1_ parameters are set too low (e.g., intensity or current direction) to influence P_X_ (Valero-Cabré et al., 2017); (3) brain-intrinsic factors such as neurochemical and neurophysiological properties of A_1_ prevent an alteration in its excitability (e.g., it is impossible to facilitate or inhibit A_1_ to a greater extent than it is before rTMS_1_ application; Karabanov et al., 2015); and (4) to influence A_1_, rTMS_1_ should be applied with greater precision (e.g., based on individual functional brain images; Hannula and Ilmoniemi, 2017). Altogether, this is enough evidence to assume that I_2_ is not a stronger form of reasoning than I_1_. I_1_ and I_2_ include a hidden assumption that rTMS_1_ leads to an alteration in A_1_ activity but not all active rTMS applications have neural effects. To claim that A_1_ has changed, the assertion based on the inference presented below has to be true:

S_1A—_rTMS_1_ is applied to A_1_

A_1C—_a change in A_1_ activity is present

$${\text{I}}_{\text{3}}\left(\left({\text{S}}_{\text{1A}}\wedge {\text{A}}_{\text{1C}}\right)\wedge {\text{S}}_{\text{1A}}\right)\to {\text{A}}_{\text{1C}}$$

I_3_ states that the statement that A_1_ activity is changed if the following two premises are true: (1) a change in A_1_ activity is present if rTMS_1_ is applied to A_1_; and (2) rTMS_1_ is applied to A_1_.

The issue of the impact of rTMS_1_ on the activity of A_1_ might be addressed with the use of neuroimaging.

## TMS and Neuroimaging

A way of strengthening TMS-based inferences is to combine TMS with neuroimaging, the advantages of which have already been exhaustively described (e.g., Sack, 2006; Bestmann et al., 2008b; Bergmann et al., 2016). Multiple studies have already successfully employed neuroimaging to determine whether a particular rTMS protocol leads to a change in A_1_ activity (e.g., Bestmann et al., 2008c; Ruff et al., 2008; Capotosto et al., 2012). Despite the advantage of neuroimaging methods in allowing detection of a change in A_1_ activity, confirmation that the change in A_1_ activity accompanies TMS_1_ cannot fully confirm H. Importantly, even if the change in A_1_ activity can be confirmed with neuroimaging, it does not always lead to a difference in T_X_ performance (Reithler et al., 2011). TMS_1_ may have no observable effect because: (1) TMS_1_ could have additional consequences that hinder the original stimulation effect, such as the occurrence of compensatory effects that diminish the TMS-induced alteration in A_1_ activity or that fulfill the function of A_1_ (Andoh and Martinot, 2008); and (2) T_X_ may not provide an adequate measure of P_X_ because T_X_ or its performance level is not demanding enough to be influenced by TMS_1_, or T_X_ is not sensitive enough to capture the impact of TMS_1_. Nevertheless, this does not imply that null TMS results are not meaningful because they are crucial to proving the functional irrelevance of a brain region to performing a particular function (de Graaf and Sack, 2011).

Next, assume that the influence of TMS_1_ on A_1_ can be effectively measured by neuroimaging methods and T_X_, and both a change in A_1_ activity and a difference in T_X_ performance is observed. This leads to stronger reasoning than I_1_ (inference 4; I_4_):

H – P_X_ takes place in A_1_

S_1A—_rTMS_1_ is applied to A_1_

T_XD—_a difference in T_X_ performance is observed (as compared to a control condition)

A_1C—_a change in A_1_ activity is present

$${\text{I}}_{\text{4}}\left(\left(\left(\left(\text{H}\wedge {\text{S}}_{\text{1A}}\right)\to {\text{T}}_{\text{XD}}\right)\wedge \left({\text{S}}_{\text{1A}}\wedge {\text{T}}_{\text{XD}}\right)\right)\left(\left(\left({\text{S}}_{\text{1A}}\to {\text{A}}_{\text{1C}}\right)\wedge {\text{S}}_{\text{1A}}\right)\to {\text{T}}_{\text{XD}}\right)\right)\to \text{H}$$

I_4_ states that the statement that P_X_ takes place in A_1_ is true if the following two premises are true: (1) the antecedent of I_1_; and (2) a difference in T_X_ performance is observed if the antecedent of I_3_ is true (analogous reasoning including ⇁ T_XD instead of T_XD_ can be used to infer about the lack of A_1_ involvement in P_X_).

Again, since the inference is inductive, I_4_ is not immune to error and H might be false. Even if it is not, I4 merely adds to I_1_ that whenever rTMS_1_ is applied to A_1_, its activity is changed, and if this occurs then a difference in T_X_ performance is observed. However, this reasoning pattern does not guarantee the correctness of the conclusion that the change in A_1_ activity is a cause of the difference in T_X_ performance, and therefore that P_X_ takes place in A_1_. It may be the case that TMS_1_ is a cause of both the change in A_1_ activity and the difference in T_X_ performance, but the change in A_1_ activity is not a cause of the difference in T_X_ performance. Thus, the causal inference between rTMS_1_ to A_1_ and the difference in T_X_ performance is stronger when the purported cause is brain stimulation but not when the purported cause is the change in brain activity, i.e., TMS causes are not analogs of neural causes. To strengthen I_4_ inference one might additionally provide evidence that whenever the difference in T_X_ performance is observed the change in A_1_ activity is present (inference 5; I_5_):

H – P_X_ takes place in A_1_

S_1A—_rTMS_1_ is applied to A_1_

T_XD—_a difference in T_X_ performance is observed (as compared to a control condition)

A_1C—_a change in A_1_ activity is present

$${\text{I}}_{\text{5}}\left(\left(\left(\left(\left(\text{H}\wedge {\text{S}}_{\text{1A}}\right)\to {\text{T}}_{\text{XD}}\right)\wedge \left({\text{S}}_{\text{1A}}\wedge {\text{T}}_{\text{XD}}\right)\right)\wedge \left(\left(\left({\text{S}}_{\text{1A}}\to {\text{A}}_{\text{1C}}\right)\wedge {\text{S}}_{\text{1A}}\right)\to {\text{T}}_{\text{XD}}\right)\right)\wedge \left({\text{T}}_{\text{XD}}\to {\text{A}}_{\text{1C}}\right)\right)\to \text{H}$$

I_5_ states that the statement that P_X_ takes place in A_1_ is true if the following two premises are true: (1) the antecedent of I_4_; and (2) a change in A_1_ activity is present if a difference in T_X_ performance is observed.

I_4_ and I_5_ are improvements over I_1_, and I_2_ and provide more confidence in TMS results. However, the limits of TMS-based conclusions also strongly depend on the complexity of the brain processes/cognitive functions investigated. The assumption that P_X_ takes place in A_1_ may be simply inadequate because the complexity of P_X_ may require it to be executed by a network rather than a single area (Pessoa, 2014), i.e., a brain area determined with TMS to be “responsible” for a certain cognitive function may be necessary but not sufficient for the realization of this cognitive function. Thus, instead of focusing on the functional properties of a single brain area, often it is necessary to investigate the functional interactions between remote but interconnected brain regions (for a review of different paradigms, see Romei et al., 2016). However, even though H might alternatively state that A_1_ is partly (not fully) responsible for P_X_, all the above issues related to the described inferences still hold.

In essence, the employment of neuroimaging may allow the following questions to be answered: (1) Does rTMS_1_ applied to A_1_ lead to a detectable change in A_1_ activity (Siebner et al., 2000)?; (2) How big is the influence of rTMS_1_ on areas adjacent to A_1_?; (3) Which areas are functionally connected to A_1_, and are they involved in P_X_ and/or T_X_ (Bestmann et al., 2005)?; (4) How does rTMS_1_ affect connectivity between certain brain areas or networks (Gratton et al., 2013)?; (5) What is the relation between the effects of rTMS_1_ and the other brain activations that occur during T_X_?; (6) What is the relation between the effects of rTMS_1_ and the difference in T_X_ performance?; and (7) Which kind of neuroplastic changes arise, and when (Poeppl et al., 2018)? These investigations might be supported by the use of effective connectivity measures (Iwabuchi et al., 2019) based on the application of causal dynamic modeling, Granger causality (Friston et al., 2013), or graph theory (Farahani et al., 2019). Additionally, novel modeling approaches that can localize cortical TMS effects might be employed to determine whether the cortical area is effectively stimulated by TMS (Weise et al., 2020). At the same time, neuroimaging evidence can include confounding activations rather than clearly represent the network responsible for the cognitive function X because: (1) TMS_1_ may serve as a common cause that has several transcranial and non-transcranial consequences (Conde et al., 2019), thus some of the brain activations (including compensatory mechanisms) may be unrelated to P_X_; and (2) engagement in T_X_ may activate processes unrelated to P_X_ (which can be addressed with appropriate control conditions). Therefore, determining whether observed changes in brain activity are associated more with activity change in A_1_ or its adjacent areas and differentiating between network effects related to P_X_ and compensatory effects is both challenging. In sum, the above patterns of reasoning may still lead to false conclusions, especially if no adequate control condition is employed.

## rTMS Control Conditions

TMS might result in various psychological, auditory, and somatosensory side effects that might trigger shifts of attention, influence alertness, or interact with elements of the experimental task. Factors like the placement of the TMS coil or the occurrence of a clicking sound can influence task performance. For example, Duecker et al. (2013) showed that lateralized sham TMS pulses caused automatic shifts of spatial attention towards the location of the TMS coil. The use of sham TMS is intended to account for the impact of active TMS’s placebo and sensory side effects. The former is related to behavioral and cognitive changes (including certain expectations) that result from a person’s belief that their brain is being stimulated, while the latter is related to somatosensory effects (e.g., muscle twitches), peripheral nerve stimulation, and auditory effects (perception of a clicking sound). The sham approach might induce placebo effects of different magnitude (Burke et al., 2019). The mismatch between active TMS and the sensory effects of control TMS can form participants’ beliefs about the effectiveness of brain stimulation. The sham approaches can to a certain degree reproduce the sensory effects of active TMS without meaningfully influencing brain activity. They are based on the employment of either regular but tilted TMS coils, in which case, the electric field can still be sufficiently strong to result in somatosensory effects and peripheral nerve stimulation (Loo et al., 2000; Lisanby et al., 2001) or purpose-built sham TMS coils which have a magnetic shield that attenuates the electromagnetic field and prevents stimulation of the brain concurrently limiting somatosensory and peripheral nerve stimulation effects (for a review, see Duecker and Sack, 2015). To mitigate the trade-off between invoking somatosensory effects and not stimulating the brain, Duecker and Sack (2015) recommend the use of surface electrodes for skin stimulation in combination with a sham TMS coil.

However, sham TMS approaches do not demonstrate area specificity. Thus, Duecker and Sack (2015) recommend it might be beneficial to use sham TMS over each brain area where active TMS is applied to ensure that all stimulation sites have a control condition for the sensory side effects of TMS. Proper choice of control condition/s involves taking into account the difference between clinical and experimental research as well as whether and how the investigated process can be influenced by participants’ beliefs. While single-blinding seems to be feasible in between-subject designs, due to distinctive TMS effects, double-blinding is difficult to perform (Broadbent et al., 2011). However, it is practiced to use the sham and active TMS coils that are indistinguishable to the researcher carrying out the stimulation, and/or this researcher is not informed about the hypothesis of the study (Basil et al., 2005). One might also minimize the placebo effect-related issues by the employment of between-subject designs (on the cost of increasing interindividual variability). Despite the chosen design, the researcher might gather from participants information on blinding success or how the TMS was experienced in a form of a short questionnaire which can further inform the study results (Flanagan et al., 2019). An alternative to the control stimulations (including active and sham TMS control strategies) might be an investigation of interindividual differences in the response to TMS measured with neuroimaging techniques and correlating them with the chosen behavioral measure.

The probabilistic strength of inferences based on experimental studies largely depends on the type of control condition used. Below, we discuss how considerations regarding control condition/s apply to TMS research designs. In general, when investigating whether P_X_ underlies cognitive function X, the simplest study designs consist of investigating a difference in T_X_ performance between pre-and post-TMS conditions or between the application of TMS1 and a sham rTMS protocol (rTMS_0_) to the same area (Duecker and Sack, 2015).

Suppose that TMS_1_ ab rTMS_0_ protocols were applied to A_1_. If a difference in TX performance is observed between rTMS_1_ and rTMS_0_ conditions, besides explanations based on sensory and placebo TMS effects (Duecker and Sack, 2015) there are alternative explanations that should be taken into consideration that is related to the direct and indirect influence of TMS on: (1) the areas surrounding A_1_; (2) excitability of A_2_, which could be more important for executing P_X_; (3) processes responsible for general cognitive functions; and (4) processes not specific to cognitive function X but to T_X_ execution. Given this, eliminating these possible alternative explanations should guide the designs of TMS studies.

### Protocol Control

Ideally, rTMS_0_ should account for sensory and placebo effects of rTMS_1_ but does not cause a change in A_1_ activity (Duecker and Sack, 2015). Typically used rTMS_0_ that attempts not to influence brain activity fail to control for all the effects that are not specific to the change in A_1_ activity because we might assume the ideal control should influence areas which are stimulated when A_1_ is targeted with TMS to separate the consequence of the change in A_1_ activity from the consequences of influencing other brain areas. For example, if an area is embedded in brain folds or lies relatively deep in the brain, then distal cortical areas which are situated above that area are affected by the electrical field, most likely more strongly (Heller and van Hulsteyn, 1992). This issue (a direct stimulation influence on the areas surrounding A_1_) can be partly addressed with a control condition by diminishing the intensity of the used protocol to account for the stimulation of the areas lying above A_1_, i.e., influencing cerebrospinal fluid distribution or superior areas while not reaching A_1_ in a significant manner. However, it has to be taken into account that the relationship between TMS protocol intensity and its outcome might not be linear (e.g., Chung et al., 2018). Additionally, active protocols with certain frequency patterns are often classified in TMS literature as “inhibitory” or “excitatory”. Thus, sometimes the protocol patterns of rTMS_1_ and another active rTMS protocol 2 (rTMS_2_) differ and might be commonly conceived as being inhibitory and excitatory, respectively; thus, they are used to obtain a difference in T_X_ performance directly (e.g., Gann et al., 2020) or to prime cortex excitability before the application of other protocols (e.g., Todd et al., 2009). It is important to note that inhibitory and excitatory rTMS properties are extrinsic to the protocol pattern and may vary depending on, e.g., protocol length, current direction, intensity, genome, and the targeted area characteristics, including its tissue excitability history and tissue excitability before protocol application (Polanía et al., 2018). Therefore, applying TMS_1_ and TMS_2_ separately to A_1_ cannot inform what change or difference in A_1_ activity is represented by a difference in T_X_ performance unless it is previously known how the activity of A_1_ is related to the difference in T_X_ performance, or the change in A_1_ activity was recorded with neuroimaging methods that can differentiate between an increase or a decrease of A_1_ activity.

### Area Control

The following, previously mentioned, issues can be addressed with a control condition that includes a control area: (1) stimulation of areas next to A_1_; (2) an indirect network effect on A_2_ activity that is more important for executing P_X_; and (3) influence on processes responsible for more general cognitive functions than cognitive function X issue that undermine the strength of TMS-based inferences. In TMS studies, it is often assumed that an adequate control condition employs a stimulation protocol that affects an area that has the lowest possibility of playing a role in P_X_ or does not influence the brain at all.

For a long time, the vertex was conceived to be such a site because it was presumed that its stimulation does not affect the brain at all. Nonetheless, several years ago it was shown that the blood oxygen level-dependent (BOLD) signal decreases in the default mode network after applying 1 Hz rTMS to the vertex, and this is not accompanied by any significant BOLD increases throughout the brain (Jung et al., 2016). The authors concluded that this supports the use of vertex simulation as a control condition. However, such a conclusion is problematic for several reasons. First, it presumes that an increase in the BOLD signal, which determines which parts of the brain are most active, will be observed after the application of a protocol that predominantly acts in an inhibitory manner (Fitzgerald et al., 2002). Second, there is an assumption that a decrease in the BOLD signal cannot indicate a change in neuronal activity (which could represent an increase in the activity of inhibitory neurons). Also, distinctly increasing and decreasing neuronal activity in an area is not equivalent to improving and impairing a cognitive function that depends on this area. Some brain processes require a decrease in local brain activity, e.g., deactivation has often been observed in the hippocampus during encoding and retrieval tasks believed to recruit this brain structure (Axmacher et al., 2009). Third, there is an assumption that the adequate control area is the one with the lowest possibility of affecting P_X_. Targeting A_2_ (an area which is not anticipated to carry out P_X_) does not confirm the specificity of A_1_ for carrying out P_X_, i.e., that P_X_ is carried out exclusively in A_1_. Since the evidence in favor of the specificity of A_1_ is based on inductive reasoning, in theory, it would be required to effectively stimulate all brain areas to conclude that A_1_ and only A_1_ is responsible for P_X_. Conceivably, an opposite approach should be adopted: adequate control for the site requires the selection of a control site that has a high probability of influencing P_X_. However, this approach is challenged by consideration of possible indirect network influences on A_1_ due to the possibility of the control site’s involvement in processes interacting with P_X_. Furthermore, assume that P_X_ requires activation in areas A_1_ and A_2_. When a difference in T_X_ performance between the conditions with rTMS_1_ to A_1_ and rTMS_1_ to A_2_ is analyzed and rTMS_1_ in the first condition resulted in impairment of T_X_ performance but in the second condition resulted in improvement of T_X_ performance, one might erroneously conclude that only one area is crucial for X. Similarly, if rTMS_1_ in both conditions influenced T_X_ performance in the same manner, one might erroneously conclude that rTMS_1_ was ineffective. Thus, limiting control conditions to area control might be not sufficient to adequately explain the TMS effect.

### Task Control

The issues of influencing processes responsible for more general cognitive functions rather than cognitive function X and influencing processes specific to T_X_ but not to cognitive function X, both of which weaken the strength of TMS-based inferences, can be addressed with task control. Dissociations may help reduce the probability of drawing erroneous conclusions on the neural bases of cognitive functions (Machery, 2012). To solve complex issues regarding certain cognitive functions or to include a task control condition in a study, e.g., to demonstrate that a certain brain area is selectively engaged in the execution of P_X_ but not in the execution of the neuronal process that underlies a different cognitive function Y (P_Y_), rTMS can be employed to determine whether the neural underpinnings of cognitive functions X and Y differ. In this case, inferences can be based on a single dissociation that is observed whenever TMS influences T_X_ and influences T_Y_ to a lesser extent. This may lead to the conclusion that A_1_ plays a role in P_X_ but not P_Y_.

**Figure 2:**
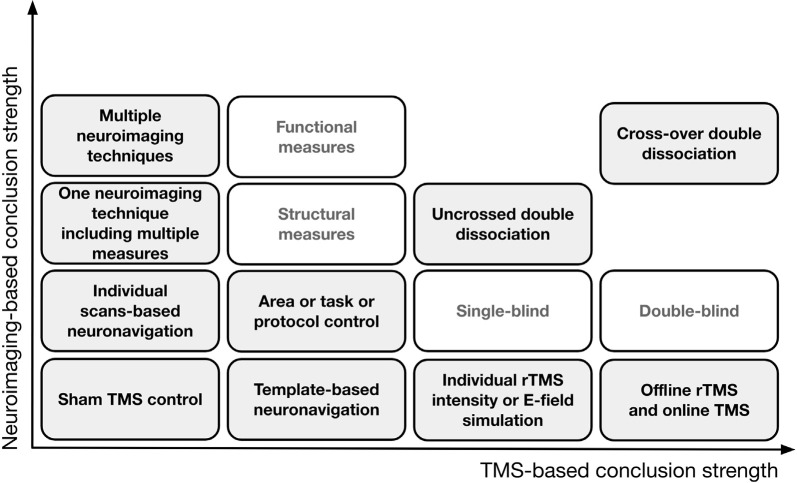
A theoretical representation of the solutions that aim to control for confounds in TMS research and improve the logical strength of the premises behind the conclusions. On the horizontal axis, progress in TMS-based conclusion strength is depicted when only TMS is employed; the vertical axis represents conclusion strength when neuroimaging is involved.

However, the results of studies employing task control may still be confounded by the confounds already mentioned. Additionally, the following confounds might be present: (1) a task that taps into one of two processes (T_X_ into P_X_) might be less sensitive than a task that taps into another one (T_Y_ into P_Y_); (2) due to its characteristics, P_X_ might be more difficult to measure than P_Y_; (3) the relative difficulties of T_X_ and T_Y_ are likely to require a different amount of available cognitive resources (e.g., memory, attention); (4) when cognitive resources are limited, different brain networks may be engaged in T_X_ or T_Y_ execution than when they are available; and (5) a discrepancy between how T_X_ and T_Y_ engage A_1_ and A_2_ can be observed, even when they recruit the same area or network, e.g., carrying out T_X_ may require a decrease in A_1_ activity, while carrying out T_Y_ may require an increase in A_1_ activity. In all the above circumstances, it would be erroneous to conclude with certainty that cognitive functions X and Y are based on two distinct brain substrates. The solution may consist of designs that combine different control approaches and allow double dissociation (Dunn and Kirsner, 2003), e.g., T_X_ but not T_Y_ performance is impaired when rTMS_0_ application and rTMS_1_ application outcomes are compared after stimulation to A_1_, while T_Y_ but not T_X_ performance is impaired when the rTMS_0_ and rTMS_1_ outcomes are compared after stimulation to A_2_. In the case of an uncrossed double dissociation, a difference in T_X_ performance and a difference in T_Y_ performance is observed when A_1_ condition and A_2_ condition are compared (when pre-and post- rTMS_1_ or rTMS_1_ and rTMS_0_ are compared) but one condition is associated with higher performance in both tasks. A cross-over double dissociation is observed when rTMS_1_ to A_1_ influences T_X_ performance more than rTMS_1_ to A_2_, and rTMS_1_ to A_2_ influences T_Y_ performance more than rTMS_1_ to A_1_ (for a summary of the solutions that aim to control for TMS confounds, see Figure 2).

Can it then be concluded that P_X_ takes place in A_1_ while P_Y_ takes place in A_2_? Unfortunately, most of the mentioned confounds also apply to double dissociations (e.g., rTMS_1_ to A_1_ reduces the available cognitive resources to T_X_, while S_2_ to A_2_ reduces them to T_Y_). In the case of uncrossed double dissociations, the additional confound may be that the task demand function for A_1_ increases monotonically, while the task demand function for A_2_ is U-shaped: A_2_ is more active when a task requires fewer or more cognitive resources. In such circumstances, if T_X_ and T_Y_ recruit a single process whose neural correlate includes A_1_ and A_2_, for A_1_ the greater task demands may correspond to the increase in its activity, while for A_2_ the greater task demands can correspond to its inactivation. Such an issue can be avoided when a cross-over double dissociation is observed, but the following confounds may still be present: (1) neuroplasticity-like effects occur at a different rate in A_1_ and A_2_ (e.g., depending on the type of brain cells affected by the stimulation); (2) rTMS_1_ and rTMS_2_ protocols applied to different areas may differently influence excitability in these areas; (3) an increase in A_1_ excitability results in a decrease in A_2_ activity, which is necessary to perform T_Y_, while an increase in A_2_ excitability results in inactivation of A_1_, which is the area necessary to perform T_X_; (4) the execution of P_X_ may correspond to A_1_ activity increase while the execution of P_Y_ may correspond to A_1_ inactivation; and (5) both A_1_ and A_2_ are recruited depending on the available cognitive resources, and the processes recruited when the amount of available resources is greater differ from the processes recruited when fewer resources are available. In all the above circumstances, it would be premature to conclude with certainty that cognitive functions X and Y are based on two distinct brain substrates.

In certain types of research (mostly preclinical and clinical studies), rTMS effects might be studied using longitudinal designs. The effect of longitudinal rTMS studies can be long-lasting, thus they can be used to investigate stable neuroplastic changes and determine whether the observed rTMS effect consistently arises over the time course of a study (Auriat et al., 2015). They also reduce the erroneous identification of side effect-associated changes as the brain stimulation effect, and they enable the employment of multiple testing measures. Similar to single-session rTMS effects, the rTMS effects in longitudinal studies might be related to individual excitability of brain areas, but they are less prone to the influence of day-to-day fluctuations in cortex excitability (Huber et al., 2013). However, there is still a possibility that the long-term effects of neuroplasticity in longitudinal studies might be related to placebo effects or be influenced by confounding factors that occur over the time course of the study.

## Conclusions

TMS has traditionally been used to provide evidence for functional brain specialization. Nevertheless—as has been getting clearer over the past two decades—the application of rTMS alone does not allow causal inferences to be drawn on neural causes without additional assumptions. A change in the execution of an experimental task might be a consequence of rTMS but at the same time not a consequence of a change in the excitability of a targeted area. However, this might be avoided when: (1) the research question is grounded in previous research and accounts for the complexity of the investigated cognitive function; (2) neuroimaging/neurophysiological techniques are employed to monitor the direct and indirect influence of rTMS; and (3) more than one control condition is employed in a single experiment to reduce the number of possible interpretations. On one hand, functional neuroimaging could make it possible to determine whether the process responsible for the investigated cognitive function has local or network characteristics and can be used to study the spread of TMS effects throughout the brain networks. On the other hand, confounding factors of neuronal correlates of investigated cognitive processes need to be addressed within each TMS-neuroimaging study. Although TMS has been proven to be a very effective brain stimulation method, its characteristic features have to be considered in reasoning based on its employment. In this article, we have clarified the difference between the causal effects of TMS and structure-related causal effects, and we have pointed out that the latter can be divided into direct and network effects. We have also outlined issues related to TMS-based inferences. Taking them into account requires limiting the extent of TMS-based reasoning but at the same time may support analysis of possible confounds and improve research designs to alleviate these confounds. Although the aforementioned issues are often addressed by experts in the field of non-invasive brain stimulation, we hope that the presented summary and theoretical analysis will help researchers who are developing the field of human-neuroscience based on TMS-based inferences. Even though rTMS without neuroimaging cannot unequivocally prove structure-related causal claims concerning direct relations between brain processes carried out in certain areas and certain behaviors/cognitive functions, it might be used for probabilistic statements about causal influences if its limitations are kept in mind. The fact that combining rTMS with neuroimaging techniques allows stronger inferences to be made does not imply that one should use rTMS only in combination with neuroimaging or/and multiple control conditions. The need for neuroimaging or/and multiple control conditions depends on the research question guiding the study and how its results are intended to be interpreted. There is a trade-off between the inferential limit and experimental feasibility; therefore, when feasible, combining rTMS with neuroimaging, multiple control conditions, and/or perturbational TMS is recommended and might provide further support for conclusions regarding experimental outcomes.

## Author Contributions

JH drafted the manuscript. MK, KS, and MW suggested changes and provided comments on the manuscript. JH improved the manuscript. All authors contributed to the article and approved the submitted version.

## Conflict of Interest

The authors declare that the research was conducted in the absence of any commercial or financial relationships that could be construed as a potential conflict of interest.

## Abbreviations

A_1C_: a change in A_1_ activity is present
BOLD: blood oxygen level-dependent
H: P_X_ takes place in A_1_
P_X_: process underlying cognitive function X
P_Y_: process underlying cognitive function Y
I_1_: inference 1
I_2_: inference 2
I_3_: inference 3
I_4_: inference 4
I_5_: inference 5
rTMS: repetitive Transcranial magnetic stimulation (TMS)
S_0_: a sham rTMS protocol
rTMS_1_: an active rTMS protocol 1
S_1A_: rTMS_1_ is applied to A_1_
rTMS_2_: an active rTMS protocol 2
TMS: transcranial magnetic stimulation
T_X_: task X
T_XD_: an observed difference in T_X_ performance
T_Y_: task Y.
